# Role of protein conformation and weak interactions on γ-gliadin liquid-liquid phase separation

**DOI:** 10.1038/s41598-019-49745-2

**Published:** 2019-09-16

**Authors:** Line Sahli, Denis Renard, Véronique Solé-Jamault, Alexandre Giuliani, Adeline Boire

**Affiliations:** 1 INRA, UR1268 Biopolymers Interactions Assemblies, 44300 Nantes, France; 2 DISCO beamline, Synchrotron Soleil, l’Orme des Merisiers, 91192 Gif sur Yvette, France; 3 UAR 1008, CEPIA, INRA, BP 71627, F-44316 Nantes, France

**Keywords:** Proteins, Intrinsically disordered proteins

## Abstract

Wheat storage proteins, gliadins, were found to form *in vitro* condensates in 55% ethanol/water mixture by decreasing temperature. The possible role of this liquid-liquid phase separation (LLPS) process on the *in vivo* gliadins storage is elusive and remains to be explored. Here we use γ-gliadin as a model of wheat proteins to probe gliadins behavior in conditions near physiological conditions. Bioinformatic analyses suggest that γ-gliadin is a hybrid protein with N-terminal domain predicted to be disordered and C-terminal domain predicted to be ordered. Spectroscopic data highlight the disordered nature of γ-gliadin. We developed an *in vitro* approach consisting to first solubilize γ-gliadin in 55% ethanol (v/v) and to progressively decrease ethanol ratio in favor of increased aqueous solution. Our results show the ability of γ-gliadin to self-assemble into dynamic droplets through LLPS, with saturation concentrations ranging from 25.9 µM ± 0.85 µM (35% ethanol (v/v)) to 3.8 µM ± 0.1 µM (0% ethanol (v/v)). We demonstrate the importance of the predicted ordered C-terminal domain of γ-gliadin in the LLPS by highlighting the protein condensates transition from a liquid to a solid state under reducing conditions. We demonstrate by increasing ionic strength the role displayed by electrostatic interactions in the phase separation. We also show the importance of hydrogen bonds in this process. Finally, we discuss the importance of gliadins condensates in their accumulation and storage in the wheat seed.

## Introduction

Liquid-liquid phase separation (LLPS) of disordered or partially disordered proteins emerges as a widespread phenomenon with broad implications for cell physiology^1–3^. These singular protein condensates result of dynamic association of protein lacking well defined 3D structure^4–7^. Their association can lead to formation of membrane-less compartments necessary for the intracellular space organization and the segregation of biochemical reactions^8–10^. So far, many types of membrane-less organelles in plants, with high prevalence of intrinsic disordered proteins (IDPs), have been reported^11^. Pyrenoid^12–14^ and photobodies^15^, respectively located in chloroplast and nucleus, are best-known plant-specific membrane-less compartments, containing proteins with high disorder profiles. Recent *in vitro* studies suggest that LLPS could also drive membrane-enclosed organelles formation in wheat seed^16,17^. It has been hypothesized that condensates of wheat storage proteins might play a role as precursor in the formation of their storage organelles, also called protein bodies (PBs).

Wheat seed contains storage proteins, prolamins, which are synthetized and accumulated into the rough endoplasmic reticulum before being deposited into PBs^18–20^. These PBs correspond to highly dense spherical organelles, surrounded by a limiting membrane, able to fuse subsequently by coalescence^19,21,22^. Mechanisms involved in their biogenesis and their organization are still unknown^23^. Remarkably, all prolamins are known for their low complexity sequence containing rich interspersed repeats which can lead to disordered structures^24–26^. Previous works in aqueous buffer/ethanol solution (45/55) (v/v) have shown the ability of whole gliadins extract, comprising a mixture of α, β, γ and ω-gliadins, to self-assemble through LLPS by decreasing temperature^16,27^. It also has been demonstrated the ability of dense gliadins phases to remain in the liquid-like state, even at high protein concentrations (up to 500 g/L) and at low temperatures (from 2 to 20 °C)^16,28^. Through these results, it has been speculated that phase separation of gliadins initiates the formation of PB in the wheat seed. Consensus definition of gliadins is that they are soluble in 70–80% aqueous alcohol or at high and low pHs^29^. Due to their supposed water-insolubility, few studies of gliadins behavior in 100% aqueous media have been done until now. In order to better understand mechanisms underlying PBs formation, a study in more relevant biological conditions is necessary.

In our study, we aim to better understand mechanisms involved in the storage and the compact organization of wheat proteins in PBs. We therefore investigate the behavior of a purified storage model protein, γ-gliadin, starting from a mixed solvent (ethanol aqueous solvent) where the protein is soluble to an aqueous solvent in order to be closer to physiological conditions found in PBs^30^. γ-gliadins comprise various isoforms denominated from their electrophoretic mobility^31,32^. In order to avoid this molecular diversity, we only focus on the γ44 isoform. We show the ability of γ44-gliadin to phase separates into dynamic liquid-like droplets or condensates even in the absence of ethanol. Reduction assays highlight the critical role of the predicted 3D structure of the C-terminal domain in this phase separation. We also demonstrate that both hydrogen and electrostatic forces drive the LLPS of the protein. Finally, we discuss the importance of gliadins condensates in the formation and regulation of PBs.

## Results and Discussion

### γ-gliadin is predicted to be partially disordered

**Figure 1:**
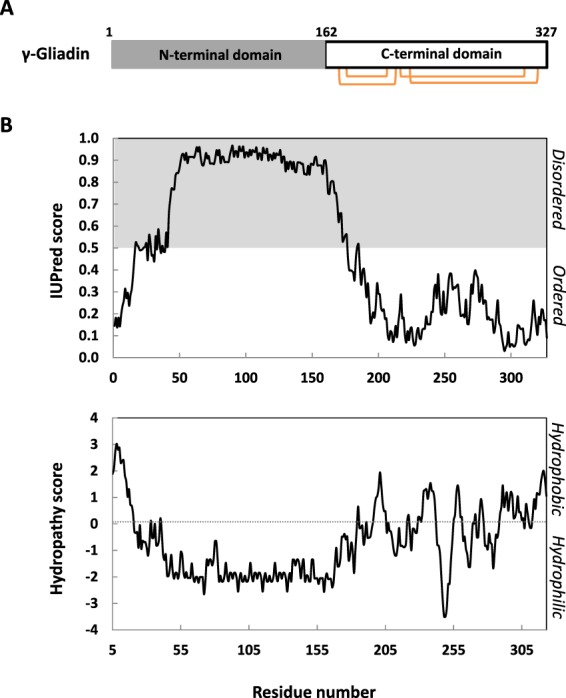
*In silico* analysis of gamma-gliadin sequence. (**A**) Schematic representation of γ-gliadin (UniprotKB-P08453) with the high repetitive N-terminal domain (grey) and C-terminal domain (white) containing four disulfide bonds (orange). (**B)** *(Top)* IUPred plot predicts the intrinsic disorder of γ-gliadin. Residues with a score above 0.5 are predicted disordered (grey area), and residues with a score below 0.5 are predicted to be ordered (white area). (*Bottom*) Kyte & Doolittle plot estimates hydropathy scores of γ-gliadin residues. Residues with positive scores are predicted hydrophobic while residues with negative scores are predicted hydrophilic.

It has been established that sulfur-rich gliadins, including γ-gliadin, contain a repetitive N-terminal domain and a non-repetitive C-terminal domain (Fig. 1A)^24–26,33,34^. From a structural point of view, bioinformatic analyses suggest that γ-gliadin comprises two distinct domains of equivalent length: one hydrophilic domain predicted to be disordered (N-terminal) and one hydrophobic domain predicted to be ordered (C-terminal) (Fig. 1B). The N-terminal intrinsic disorder is expected, since it is a highly repetitive sequence with large number of (PQQPFPQ)_*n*_ tandem repeats. Concerning the C-terminal domain, its low disorder propensity suggests less conformational flexibility, however, its hydrophobicity profile (Fig. 1B**)** could promote more energetic interactions leading to irreversible protein aggregation. Note that these predictions are consistent with results of previous spectroscopy studies. It has been demonstrated that the C-terminal domain is more rigid and less prone to conformational changes by temperature change than N-terminal domain^35^. Circular dichroism experiments showed predominance of β-turn and polyproline II helix structure in N-terminal domain of γ-gliadin while its C-terminal domain is predominantly α-helical^26,35^. Finally, γ-gliadin is expected to be an hybrid protein, divided into two domains with two different physicochemical behaviors: one flexible and dynamic non-globular domain and one rigid and stable globular domain. The C-terminal domain is known to be a more stable domain due to its four intramolecular disulfide bonds, essential to the well-defined tertiary structure^36^. Contrary to the C-terminal domain, the absence of strictly deterministic 3D structure of the N-terminal domain provides an enhanced flexibility and could allow a wide range of conformational states. This conformational plasticity could therefore promote interaction with many different protein partners and enhance assembly of more complex systems^37,38^.

### γ44-gliadin reversibly phase separates *in vitro*

**Figure 2:**
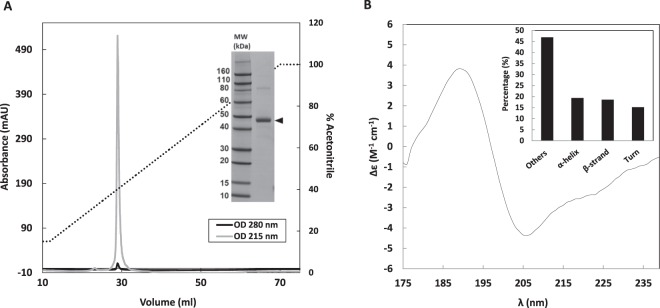
Characterization of γ44-gliadin. (**A)** Chromatographic profile of γ44-gliadin at 215 and 280 nm, eluted with acetonitrile gradient (15–100%) containing 0.06% TFA. SDS-PAGE of γ44-gliadin (black arrow at ~44 kDa) revealed by Instant blue staining. (**B)** Far-UV CD spectrum of γ44-gliadin at 51.7 μM in 0.05 M MOPS pH 7.2, 25 mM NaCl and 55% ethanol (v/v). The inset shows secondary structures content from the deconvolution of the spectrum using BestSel software^70^ (19.4% α-helix, 18.6% β-strand, 15.2% turn and 46.9% unordered structures (PPII, random coil)).

Previous works have shown the ability of gliadins isolate (α, β, γ and ω-gliadins) to phase separate into condensed liquid droplets by decreasing temperature in water/ethanol solution (45/55) (v/v)^16,27^. In the present study, γ44-gliadin behavior under conditions that tends to physiological conditions during wheat seed development (aqueous buffer, pH 7.2) is under consideration. The characterization of γ44-gliadin is first reported. γ44-gliadin protein content is of 90.5% (±1.5) on a dry basis, using the Dumas method. Reverse-phase chromatographic analysis displays an elution of γ44-gliadin between 37% and 40% of acetonitrile (Fig. 2A). Molecular weight of 38 655 Da has been established by mass spectometry (data not shown) while SDS-PAGE electrophoresis gives an apparent molecular weight of 44 000 Da (Fig. 2A). In polyacrylamide gel electrophoresis, such anomalous mobility is often observed with disordered protein because of abnormally low binding of SDS to hydrophilic sequences^39^. Hydrodynamic radius of disordered protein is higher than those of ordered protein, causing retarded mobility and over-estimation of molecular weight^40^. The electrophoretic profile appears to be another element to attest the conformational singularity of γ44-gliadin. It should be noted that the characterization of extracted and purified γ44-gliadin shows a high protein purity rarely obtained because of gliadins polymorphism. To estimate the secondary structure content of the protein, synchrotron radiation circular dichroism spectrum in far ultraviolet region was recorded (Fig. 2B). Spectral deconvolution of γ44-gliadin spectrum using Bestsel software^40^ shows a high content of unordered structure (46.9%), result consistent with bioinformatic data.

**Figure 3:**
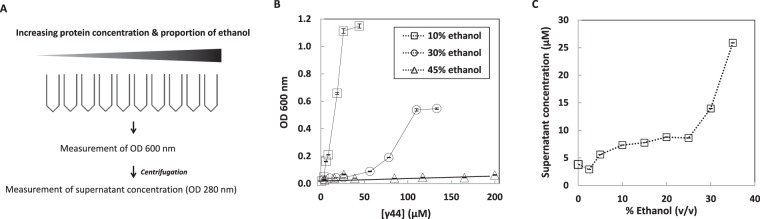
Determination of saturating concentrations by absorbance measurements. (**A)** An overall scheme of the two methods used to establish the γ44-gliadin diagram phase. (**B)** Absorbance measured at 600 nm of γ44-gliadin solutions as function of total protein concentration for 10%, 30% and 45% ethanol (v/v) (n = 3). Dotted lines stand for guide to the eye. (**C)** Concentration of soluble protein in supernatant after centrifugation (C_sat_) as function of % ethanol (v/v) (n = 3). Lines are guide for the eyes. All data are expressed as the mean ± standard deviation (SD).

**Figure 4:**
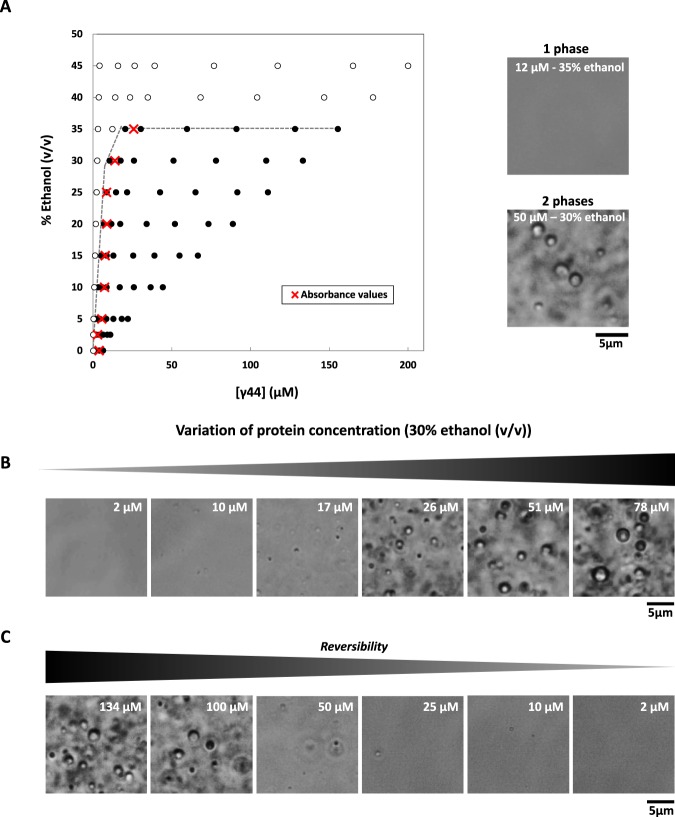
Liquid-liquid phase separation (LLPS) of γ44-gliadin with solvent perturbation. (**A)** (*Left*) Non-equilibrum phase diagram of γ44-gliadin (% ethanol (v/v) versus total protein concentration established in 50 mM MOPS pH 7.2, 25 mM NaCl. White circles represent translucid phase and black circles turbid phase. Red crosses represent saturating concentrations values (C_sat_) determined by OD at 280 nm. (*Right*) Microscopic observations of homogenous phase at 35% ethanol (v/v) (12 µM of total protein concentration) (*top*) and mixed phase at 30% ethanol (v/v) (50 µM of total protein concentration) (*bottom*). (**B)** Observations of LLPS by increasing total γ44-gliadin concentration (30% ethanol (v/v)). (**C)** LLPS reversibility by progressive dilution of γ44-gliadin solution into MOPS buffer, 25 mM NaCl and keeping constant ethanol concentration (30% ethanol (v/v)).

Protein samples were prepared by decreasing ethanol concentration as shown in Fig. 3A, excepted for the 0% ethanol condition where the protein has been directly dispersed in 0.05 M MOPS pH 7.2 and 25 mM NaCl. Absorbance of protein samples was measured at λ = 600 nm as function of initial protein concentration (Fig. 3B). Decreasing ethanol concentration leads to an increase of OD (optical density) at 600 nm with γ44-gliadin solutions being cloudy for 10% and 30% ethanol (v/v) while being transparent for 45% ethanol (v/v). Note that the OD at 600 nm is much higher at 10% compare to 30% ethanol (v/v) and that the value increases with initial protein concentration. Microscopic observations reveal that this increase of absorbance values is due to highly dynamic and spherical micrometric droplets (Fig. 4).

To determine saturation concentrations of γ44-gliadin (C_sat_) corresponding to equilibrium concentrations above which phase separation occurs, samples from 45% to 0% ethanol were prepared and centrifuged. Supernatant concentrations of centrifuged protein samples were determined by absorbance measurements after correction of turbidity (Fig. 3C). Estimated saturation concentrations ranged from 25.9 µM ± 0.85 µM (35% ethanol (v/v)) to 3.8 µM ± 0.1 µM (0% ethanol (v/v)) (Fig. 3C).

The non-equilibrum phase diagram of γ44-gliadin displayed in Fig. 4A has been established by combining OD at 600 nm data and microscopic observations. Saturating concentrations determined by absorbance measurements at 280 nm are overall consistent with the established phase boundary (red crosses). According to this protein quantification, the system phase separates at 35% ethanol (v/v), into a diluted and a concentrated phase (Fig. 4A). The number and the size of formed droplets increase with increasing protein concentration (Fig. 4B). Interestingly, the boundary of γ44-gliadin phase diagram which delimits the monophasic state from the biphasic state is reached below 26 mM (Fig. 4A) while a globular protein such as lyzozyme, phase separates from 5.6 mM upon temperature decrease^41^.

Liquid-liquid phase separation, contrary to aggregation or liquid-solid phase separation, is a dynamic and reversible process^1,42,43^. To check reversibility of the system, phase separation at 30% ethanol was first induced, then, a progressive dilution with the MOPS buffer while keeping constant ethanol percentage (50 mM MOPS pH 7.2, 25 mM NaCl, 30% ethanol (v/v)) was performed. Microscopic imaging shows a decrease of the number of droplets with decreasing protein concentration until a total disappearance at low protein concentration (Fig. 4C). These observations show the ability of γ44-gliadin to form reversible assemblies upon solvent quality change. Note that the reversibility of the system at 0% of ethanol was also confirmed (data not shown).

### γ44-gliadin form dynamic and permeable liquid-like droplets *in vitro*

**Figure 5:**
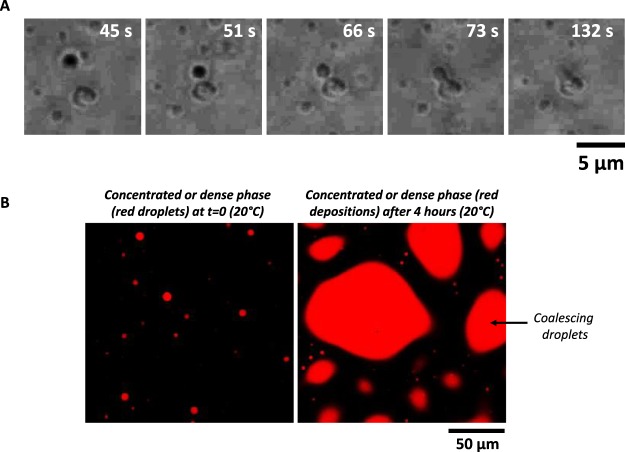
Liquid-like nature of γ44-gliadin condensates. (**A)** Phase-contrast microscopy imaging of fused γ44-gliadin droplets in time. Observation of fusion between dynamic coacervate (in black) and sedimented coacervate (in grey) was done at 46.5 µM in 50 mM MOPS pH 7.2, 25 mM NaCl and 25% ethanol (v/v). (**B)** Confocal imaging of γ44-gliadin-TRITC solution at 2 mM total protein concentration (50 mM MOPS pH 7.2, 25 mM NaCl and 25% ethanol (v/v)) before (*left*) and after 4 hours of waiting time (*right*), at room temperature (λ_ex_ ~ 555 nm and λ_em_ ~ 580 nm).

To determine the nature of γ44-gliadin droplets, observations under phase-contrast microscopy were done. γ44-gliadin forms coacervates exhibiting fusion properties (Fig. 5A). To ensure that droplets contained protein, γ44-gliadin was covalently labelled with TRITC and observed under confocal microscopy. As expected, dynamic condensates observed in the dense phase corresponds to γ44-gliadin (Fig. 5B, *left*). Further inspection after 4 H (20 °C) at the bottom of the microscope slide shows liquid deposits exhibiting irregular shape or undulating boundaries (Fig. 5B, *right*), suggesting subsequent coalescence of γ44-gliadin-TRITC droplets that settle down in time on the microscope slide. All these results demonstrate the liquid-like properties of γ44-gliadin condensates.

**Figure 6:**
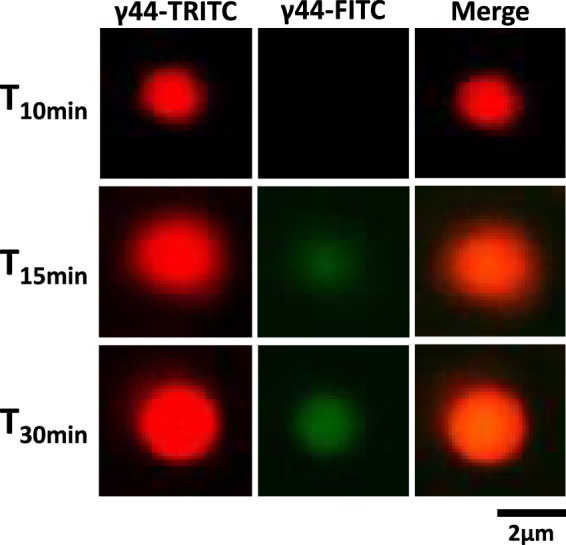
Equilibrium between the diluted and the concentrated phases in γ44-gliadin LLPS. Droplets of γ44-gliadin-TRITC were pre-formed at 20% ethanol (50 mM MOPS pH 7.2, 25 mM NaCl) after 15 min of equilibration; γ44-gliadin-FITC (molar ratio 1:1) was then added in the diluted phase (47 µM of total protein concentration). The protein diffusion assay was monitored at room temperature in time with observations made after 10, 15 and 30 min.

To determine whether γ44-gliadin concentrated phase is in equilibrium with the diluted phase, as classically defined in LLPS, protein diffusion kinetics assays were monitored using confocal microscopy. γ44-gliadin was covalently labelled using two different fluorescent dyes: TRITC (red) and FITC (green). Liquid droplets of γ44-gliadin-TRITC were first formed at 20% ethanol (v/v) after 10 min of equilibration; γ44-gliadin-FITC was then added to the sample in the continuous phase in order to see whether the protein diffuses from the diluted to the concentrated phase. To illustrate the process, one liquid droplet was chosen and followed in time (Fig. 6). Fifteen minutes after the addition of γ44-gliadin-FITC, a progressive green fluorescent signal appeared at the center of the red labelled droplet (Fig. 6). The green signal gradually diffused over time and entirely covered the red droplet resulting after merging images to an orange droplet (Fig. 6, *bottom right*). The combination of both fluorescence signals clearly demonstrates the diffusion of γ44-gliadin-FITC into pre-formed γ44-gliadin-TRITC droplets (Fig. 6). At the protein and ethanol concentrations used, diffusion from the center to the periphery of droplet took about 30 minutes. The same observations were done by starting with γ44-gliadin-FITC liquid droplets and added γ44-gliadin-TRITC (results not shown). These results highlight the dynamic behavior of liquid droplets where an equilibrium with the diluted phase is reached through free diffusion of proteins inside and outside of the droplets. However, the fluorescence intensity and the diffusion rate vary from one droplet to another. Some droplets show an early high signal intensity when γ44-gliadin-FITC is added (after few minutes) (Fig. S2).

### Electrostatic and hydrogen interactions tune γ44-gliadin phase behavior

**Figure 7:**
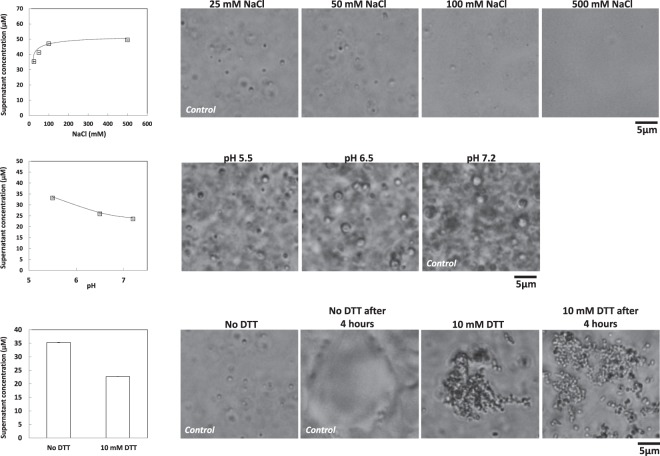
Physicochemical properties of γ44-gliadin LLPS. (**A)** (*Left*) Concentrations of γ44-gliadin at 25, 50, 100 and 500 mM of NaCl in the supernatant after centrifugation. (*Right*) Decrease of droplets number seen by phase contrast microscopy. LLPS was induced at 56 µM of total protein concentration in 50 mM MOPS pH 7.2 and 30% ethanol (v/v). (**B)** (*Left*) Concentrations of γ44-gliadin at pH 5.5, 6.5 and 7.2 in the supernatant after centrifugation. (*Right*) Increase of droplets number seen by phase contrast microscopy. LLPS was induced at 56 µM of total protein concentration and 30% ethanol (v/v) in MES buffer, at constant ionic strength (48 mM). (**C)** (*Left*) Concentrations of γ44-gliadin with or without 10 mM DTT in the supernatant after centrifugation. (*Right*) γ44-gliadin phase behavior seen by phase contrast microscopy with or without DTT before and 4 hours after adding DTT (room temperature). Phase separation was induced at 56 µM of total protein concentration in 50 mM MOPS pH 7.2, 25 mM NaCl and 30% ethanol (v/v). Plot data are expressed as the mean ± standard deviation (SD).

Electrostatic interactions are often predominant drivers for the LLPS of IDPs^8^. To determine the contribution of electrostatic forces in γ44-gliadin like droplets, assays at physiological pH (7.2) and increasing ionic strength were performed (initial protein concentration of 56 µM). Assays were carried-out at different NaCl concentrations: 25, 50, 100 and 500 mM. Increasing salt concentration led to a drastic decrease of droplets number (Fig. 7A, *right*) and to an increase of γ44-gliadin saturation concentrations (35.2 ± 1.12 µM at 25 mM NaCl and 49.4 µM ± 1.46 at 500 mM NaCl) (Fig. 7A, *left*). In other words, the presence of high salt concentration in the protein solution greatly impaired the LLPS even if some droplets were still present in solution but hardly visible under the microscope. These observations demonstrated the contribution of electrostatic interactions in the formation of γ44-gliadin liquid-like droplets. Assays at different pH values (5.5, 6 and 7.2) with constant ionic strength (48 mM) showed an increase in droplets formation with pH (Fig. 7B). The increase of pH in the medium led finally to an enhanced self-association of γ44-gliadin which translates into a decrease of the saturation concentrations (33.1 ± 0.24 µM at pH 5.5, 25.8 ± 0.35 µM at pH 6.5 and 23.5 ± 0.15 µM at pH 7.2) (Fig. 7B, *left*). A difference is observed in saturation concentrations values between the two control conditions: 35.2 µM for NaCl experiments and 23.5 µM for pH experiments (Fig. 7A,B). These observations could be explained by the different buffers used for these experiments (MOPS and MES) despite the same ionic strength (48 mM). γ44-gliadin phase behaviour with increasing ionic strength or pH is unexpected since γ44-gliadin is poorly charged^44,45^. From a physiological point of view, previous works have reported acidification in the wheat endosperm during germination to allow enzymatic hydrolysis of storage proteins^46–48^. In future works, it could be interesting to determine if the acidic pH could be another way to regulate LLPS via coacervate dissolution *in vivo* that also may regulate enzymatic digestion of gliadins.

### The predicted ordered C-terminal domain of γ44-gliadin is necessary for phase separation

Many studies have demonstrated the key role played by disordered domains in the LLPS formation^6,38,49–51^. In some works, it has also been demonstrated the contribution of ordered domain in this process^52^ *via* a synergy between ordered and disordered domains. In our case, C-terminal domain is predicted to be ordered and holds all cysteine residues of γ44-gliadin. Intramolecular disulfide bonds play a major role in the conformation of the gliadins and control the process of their deposition into protein bodies^18,36^. The contribution of C-terminal domain and its disulfide bonds in the γ44-gliadin phase behaviour was assessed by reducing its disulfide bonds. γ44-gliadin coacervates were initiated at 30% ethanol (v/v) with or without 10 mM dithiothreitol (DTT). The number of free thiol in non-reduced and reduced γ44-gliadin was determined by DTNB titration assay^53^ at 55% of ethanol (v/v). The thiol contents of the reduced γ44-gliadin (28.8 ± 0.021 mole of free thiol per mole of protein) is much higher than the non-reduced γ44-gliadin (0.1 ± 0.002 mole of free thiol per mole of protein) (Table 1, Supplementary Information). Interestingly, droplet formation followed by droplets agregation was observed under microscope, in presence of DTT (Fig. 7C, *right*). Reduction of disulfide bonds leads therefore to highly aggregated droplets, but at the same time, keep the spherical shape of droplets (Fig. 7). Note that progressive dilutions made with the same buffer confirm the irreversibility of the system (not shown). Further microscopic observations after 4 hours shows the absence of coalescence of droplets in presence of DTT, contrary to control condition, suggesting a solid-like nature of reduce γ44-gliadin droplets (Fig. 7C). This suggest the formation of solid phase instead of liquid phase. Decrease of supernatant protein concentration without (35.3 ± 1.12 µM) and with (22.6 ± 0.4 µM) reducing agent is observed (Fig. 7C, *left*). Previous circular dichroism studies have shown that reduction did not change the secondary structure of γ-gliadin^54^. All these results suggest that it is the tertiary structure of the C-terminal domain that is impaired by the disulfide bond reduction. This partial unfolding would expose hydrophobic residues which can promote protein aggregation mediated by hydrophobic interactions.

### Towards conditions close to the physiological conditions

In this work, we have demonstrated the ability of γ44-gliadin to undergo *in vitro* LLPS upon addition of aqueous solution. This phase separation process occurs at low protein concentrations compared to globular proteins, but remains in the range of IDPs saturation concentrations^7,52,55,56^. Low saturation concentrations indicate a high attraction between protein molecules. At the same time, liquid properties of droplets attest of weak interactions. We show that LLPS are initiated when ethanol proportion decreased. Hydrophobic interactions may play a critical role in the phase behaviour of γ44-gliadin as reported in other studies^1,56,57^. At the same time, the increase of the aqueous buffer proportion must promote formation of hydrogen bonds. These low energy interactions are easily dissociable, thus, compatible with liquid phase separation behavior.

**Figure 8:**
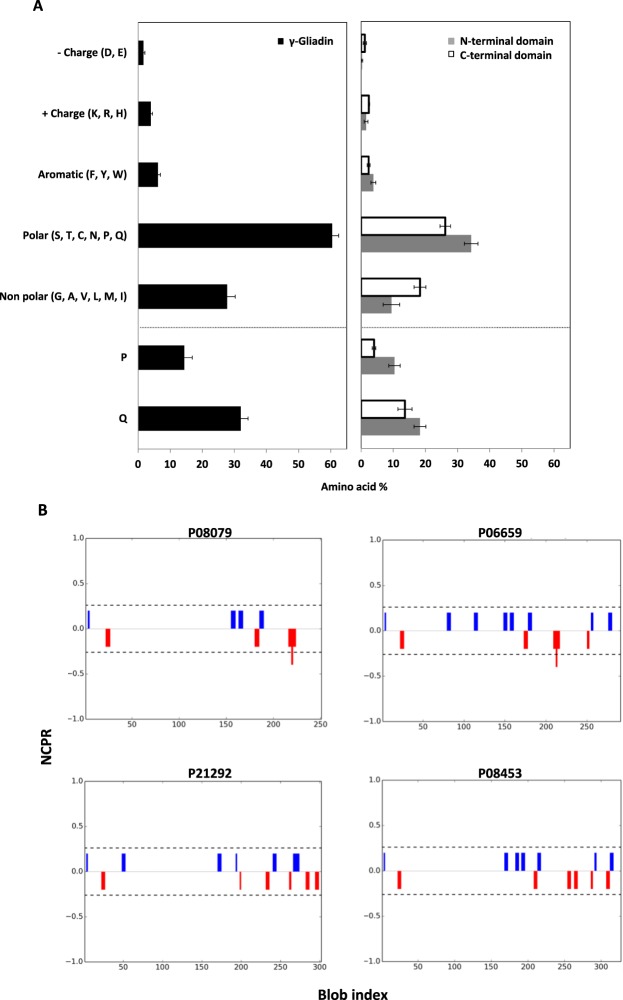
Sequence analysis of all reviewed accessions of γ-gliadin from UniprotKB. (**A)** Histograms of the amino acid composition (%) of γ-gliadin (black), N-terminal (grey) and C-terminal domain (white). Plot data are expressed as the mean ± standard deviation (SD). (**B)** Linear net charge per residue plots (NCPR) of γ-gliadin accessions from UniprotKB (P08079, P06659, P21292 and P08453) using CIDER tool^67^. Positive net charges (blue) and negative net charges (red) are represented.

We have also highlighted the contribution of electrostatic interactions in the γ-gliadin self-association despite their low-charged content. In order to explain these observations, amino acid composition of all referenced and reviewed γ-gliadins accessions from UniprotKB were analysed. γ-gliadins are poorly charged proteins, with only 3.92 ± 0.52% positive and 1.78 ± 0.43% negative charged amino acids (Fig. 8, in bla*ck*). The large net charge that characterizes IDPs^58,59^ in general is not found in γ-gliadin sequences. Interestingly, charges are mainly distributed in C-terminal domains (Fig. 8, *in white*). As expected, γ-gliadins contain high levels of Q (32.3 ± 2.28%) and P (15.93 ± 2.44%) residues which explains their original classification as prolamins^24^. N-terminal domains contain 4.28 ± 0.8% aromatic amino acids, which are mostly F residues (3.67 ± 0.26%), while C-terminal domains contain only 2.15 ± 0.44% aromatic amino acids (Fig. 8). As already established, C-terminal domains hold all cysteines residues (Fig. 1A). The N-terminal domains contain a total of 35.65 ± 2.19% polar residues against only 11.97 ± 2.4% apolar residues suggesting an hydrophilic profile. In the contrary, C-terminal domains appear more hydrophobic with 21.1 ± 1% apolar residues and 25.48 ± 1.66% polar residues. These observations are in agreement with predicted hydropathy values (Fig. 1B).

The heterogenous amino acids composition leads to an inequal charge distribution along the sequence that may promote directional interactions^60^. Indeed, aromatic residues of N-terminal domains and positively charged residues of C-terminal domains could participate to LLPS by π-cation interactions as shown recently^52,60–62^. Note that arginine residues, considered as the most important amino acids for cation-π interactions^63^, are mainly present in C-terminal domains (Fig. 8). The presence of oppositely charged amino acids in the C-terminal domains could also lead to attractive electrostatic interactions provided an uneven charge distribution within domains^64–66^. To evaluate the polarity of C-terminal domain sequences, the CIDER webserver^67^ was subsequently used. Excepted for P06659, linear net charge per residue plots of γ-gliadin accessions reveal two main groups of positive net charges clusters and two main groups of negative net charges clusters distributed in the C-terminal domain (Fig. 8B). Even if analysis seems to show the presence of patches of oppositely charged residues in C-terminal domains, the absence of γ-gliadin crystallographic data and electronic density map do not allow to validate all these observations. Electrostatic attractions would explain the salt and pH sensitivity observed in the present study. High salt concentration screens electrostatic interactions while decreasing pH causes a charge imbalance in favor of positive charges. Finally, the high abundancy of aromatic residues in the N-terminal domain might also promote protein self-association by π-π stacking interactions^5^. A summarizing figure of hypothetical interactions involved in gliadin LLPS is presented at the end of the paper.

We have shown the contribution of the C-terminal in the γ44-gliadin LLPS (Fig. 7C), the N-terminal domain may, however, also play a determining role in this process. As aforementioned, only the N-terminal domain of γ-gliadin is subjected to conformational changes upon temperature change^35^ which could contribute to gliadins LLPS upon decreasing temperature^16,27^. Experiments with truncated form of N/C terminus domain could help to elucidate more broadly their role in the plant storage proteins assembly. Further directed mutagenesis experiments are also needed to map regions or amino acid residues involved in LLPS. By this way, it could be possible to determine the type of electrostatic forces involved in the condensates formation (π-cation or charge-charge).

**Figure 9:**
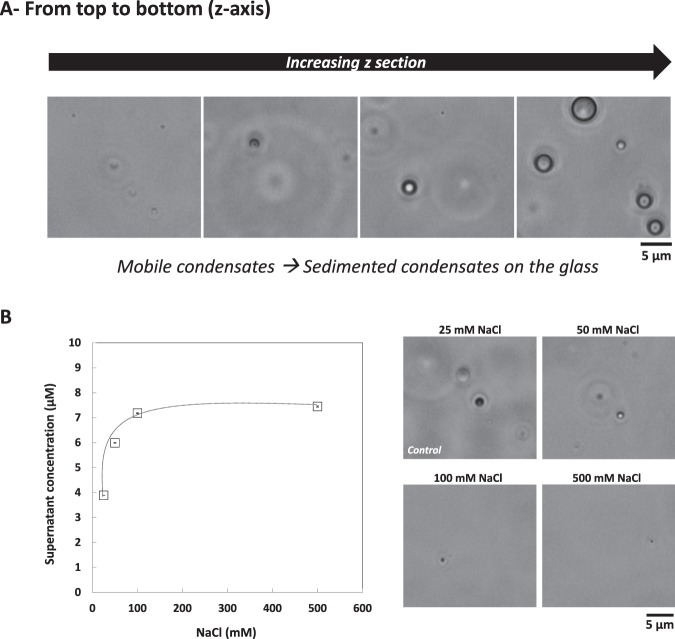
γ44-gliadin behavior under conditions close to physiological conditions (pH 7.2). (**A**) Phase-contrast microscopy imaging of γ44-gliadin at 104 µM of total protein concentration in 50 mM MOPS pH 7.2 and 25 mM NaCl. The black arrow indicates the depth of field for the observation of sample in the z-direction. (**B)** (*Left*) Concentrations of γ44-gliadin at 25, 50, 100 and 500 mM of NaCl in the supernatant after centrifugation. LLPS was induced at 104 µM of total protein concentration in 50 mM MOPS pH 7.2. Data are expressed as the mean ± standard deviation (SD). (*Right*) Decrease of droplets number imaged by phase contrast microscopy.

In the present paper, we developed an *in vitro* approach to better understand mechanisms underlying the formation and sub-organization of protein bodies *in vivo*. Our experiments with progressive decrease of ethanol proportion in the initial protein sample showed the formation of dynamic condensates of γ44-gliadin. These condensates were also observed at 0% of ethanol but in lower number (Fig. 9). These observations are explained by the spontaneous coalescence of several γ44-gliadin condensates, which results in larger droplets observed at the bottom of the microscope slide by phase-contrast microscopy (Fig. 9A). Interestingly, these sedimented condensates, still present in 25 mM NaCl, disappear with increasing ionic strength and are correlated with an increase of protein solubilization (3.8 ± 0.08 µM at 25 mM NaCl and 7.4 ± 0.63 µM at 500 mM NaCl) (Fig. 9B, *left*). The number of mobile coacervates also decrease without disappear completely (Fig. 9B, *right*). These results suggest that electrostatics interactions also contribute to γ44-gliadin LLPS in 100% aqueous media.

**Figure 10:**
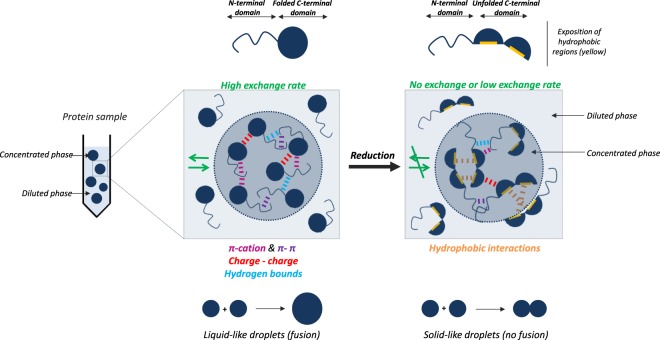
γ -gliadins contain two modules: one non-globular domain (predicted disordered) and one globular domain (predicted ordered). Electrostatic interactions tune their phase separation (π-cation/charge-charge). Hydrogen interactions are speculated also to be involved in their behavior. Liquid-like droplets are in continuous equilibrum with the dilute phase leading to a dynamic exchange of proteins between the two phases. Reduction conditions lead to the misfolding of the C-terminal domain. Protein agregates mediated by hydrophobic interactions are formed.

To conclude, a highly crowded and spherical protein environment formed by LLPS may be a good way for the wheat seed to easily accumulate mobilized proteins using minimal space (Fig. 10). The preliminary results obtained in aqueous solution (0% of ethanol) could find some plausible explanation to the *in vivo* PBs genesis where LLPS would prevail after protein biosynthesis within the endoplasmic reticulum.

## Conclusion

γ-gliadin is partially disordered and is able to self-assemble into liquid-like droplets *in vitro*. The boundary delimiting the liquid-liquid demixing zone of γ-gliadin in the phase diagram is reached at low protein concentration and shows the high attractiveness of proteins. We highlighted the contribution of hydrogen and electrostatic interactions into γ-gliadin phase condensates. It is emphasized that hydrogen bonds are largely predominant drivers for the liquid-liquid phase separation. We also demonstrated the contribution of the predicted ordered C-terminal domain in this process and the importance of its 3D conformation stabilized by disulfide bonds.

Contrary to what has been thought for a long time, γ-gliadin have the ability to form, *in vitro*, reversible, dynamic and dense molecular assemblies when conditions are shifted from good solvent conditions (55% ethanol/water mixture) to aqueous conditions found in wheat. We hypothesized that these molecular organization could be considered as a transition state or precursors leading to the formation of wheat protein bodies.

## Material and Methods

### Bioinformatic analyses

For *in silico* analyses, we focused on γ-gliadin (UniprotKB-P08453), with a molecular weight of 37 122 g/mol close to the γ-gliadin used in this study. For comparison, all referenced and reviewed protein sequences accessions found in UniprotKB with sequence identity close to 100% to γ-gliadin were also analyzed (total of four accessions) (Supplementary Information). The full length amino acid sequences were run through structural disorder predictor IUPred (http://iupred.elte.hu/). Linear net charge per residue of protein sequences were obtained using CIDER tool (https://pappulab.wustl.edu/CIDER/) and hydropathy scores by ExPASy according to Kyte & Doolittle method (https://web.expasy.org/protscale/).

### Purification and characterization of γ44-gliadin

γ44-gliadin was extracted and purified from wheat gluten of cv. Hardi as previously described^68^. Purified protein was dialysed against acetic acid (0.5 ml/L), before being freeze-dried. Protein content was determined by the Dumas method with a corrective factor of 5.7^69^. For biochemical characterisation, protein powder (150 µg) was solubilised in Tris-HCl pH 8/ethanol mixture (60/40) (v/v) and applied on an analytical column of Nucleosil C-18 (300 Å, 5 µm, 250 × 4 mm) equilibrated with deionized water containing 0.06% of trifluoroacetic acid (TFA). Elution was performed using an acetonitrile gradient containing 0.06% TFA (15–100%). Protein elution was monitored by UV absorbance at 215 and 280 nm. Protein fractions were collected, diluted once with Laemlli buffer and heated at 95 °C for 5 min for SDS-PAGE analysis (4–12% Bis-Tris Plus Gels, Bolt™). After migration, electrophoresis gel was incubated overnight in Instant blue solution to ensure gel coloration. Gel was rinsed in distilled water and scanned.

### Synchrotron radiation circular dichroism (SRCD)

Measurements were performed using the DISCO beamline at Soleil synchrotron (Gif-sur-Yvette, France). The samples were prepared at 51.7 μM in 50 mM MOPS buffer pH 7.2, 25 mM NaCl and 55% ethanol (v/v). Each spectrum is the average of three acquisitions. The spectrum of buffer was subtracted from the protein spectrum. Spectrum was smoothed using the Savitzky-Golay filtering (order 3 out of 9 points). Content of secondary structures was determined using the BestSel software (http://bestsel.elte.hu/index.php)^70^.

### Phase diagram

For assays from 45% to 2.5% of ethanol (v/v), γ44-gliadin powder was dissolved in 50 mM MOPS pH 7.2, 25 mM NaCl and 55% ethanol (v/v), under stirring, overnight, at room temperature. Residual undissolved material was removed by filtration on a 0.2 μm membrane filter (Sartorius, France). Variations of protein and ethanol concentrations were performed by adding different volumes of 50 mM MOPS pH 7.2 and 25 mM NaCl buffer. Absorbance assessments were carried out in a 96-well microplate Greiner Bio-One UV-STAR® at a wavelength of 600 nm (n = 3) using a microplates spectrophotometer (Biotek Epoch Microplate Spectrophotometer, France). Protein samples were also observed using phase-contrast microscopy (Nikon Eclipse E400, Sentech camera, France) set at the magnification of x40 to evidence the presence or absence of LLPS (n = 2). For reversibility experiments, phase separation of γ44-gliadin was induced at 30% ethanol (v/v). To probe the evolution of LLPS behavior, protein solution was progressively diluted with MOPS buffer containing 30% of ethanol (v/v) (50 mM MOPS pH 7.2, 25 mM NaCl, 30% ethanol). Microscopic imaging was monitored as previously described.

For assays at 0% of ethanol, γ44-gliadin powder was dispersed in 50 mM MOPS pH 7.2 and 25 mM NaCl, under stirring, overnight, at room temperature. Protein samples were observed by phase-contrast microscopy as previously described (n = 2).

### Determination of saturation concentrations

To determine γ44-gliadin supernatant concentrations (C_sat_), we proceeded in different ways depending on whether the sample contained ethanol or not. Samples from 45% to 2.5% ethanol were prepared with stock protein solution at 51.7 µM in 50 mM MOPS pH 7.2, 25 mM NaCl, 55% ethanol. Dilutions of protein and ethanol concentrations were done by adding increased volumes of 50 mM MOPS pH 7.2 and 25 mM NaCl buffer. Samples at 0% ethanol were prepared by dispersion of the protein powder in MOPS buffer. For separation of the diluted phase (continuous phase) from the concentrated phase (droplets), protein samples were centrifuged in eppendorf tubes (30 min, 15 000 × *g*) (Heraeus™ Primo™/Primo R, France). Liquid droplets corresponding to the dense phase were spun down. Absorbance of clarified supernatant was measured by UV absorbance using a micro-volume plate (Take 3, Biotek, USA) and a plate reader (Biotek Epoch Microplate Spectrophotometer, USA) and converted into protein concentration using an extinction coefficent of 0.55 g^−1^.L.cm^−1^ (personal data) and a molecular weight of 38 655 g/mol (determined by mass spectrometry). Note that when it was necessary, the maximum absorbance of supernatants was corrected from turbidity τ determined in the visible range by applying the relation: Log τ = a*log Abs + b. Measurements were performed in triplicate.

An estimate of C_sat_ was also performed by confocal microscopy with the measurement of fluorescence intensity outside droplets (Supplementary Information). Calibration curve of labelled γ44-gliadin, fluorescence intensity vs γ44-gliadin-TRITC concentration, was previously established at 55% of ethanol. The procedure used for confocal microscopy assays was further detailed in the “Labelling and colocalization experiment” following section. Measurements were performed in triplicate.

### Labelling and colocalization experiment

γ44-gliadin was solubilized (10 mg/ml or 258.7 µM) overnight at room temperature in 0.1 M sodium bicarbonate buffer (pH 9) containing 55% ethanol (v/v). Protein sample was then filtered (Sartorius, 0.2 µm) and incubated for covalently linking with 0.03% of fluorescein isothiocyanate (FITC) or tetramethylrhodamine (TRITC). Cross-linked reactions were done at room temperature, under gentle stirring during 1H. Free dyes were removed by dialysis protein samples (24H, 4 °C) against water/ethanol mixture (45/55) (v/v) and 50 mM MOPS pH 7.2, 25 mM NaCl, 55% ethanol (v/v). At the end, it is estimated that about one-third of proteins are bound to the fluorophore. Colocalization of γ44-gliadin-TRITC and γ44-gliadin-FITC was performed in two steps. First, droplets of γ44-gliadin-TRITC were formed at 20% ethanol (v/v) by pre-incubation at room temperature. After 15 minutes of equilibration, γ44-gliadin labelled with FITC was added to the mixture at a 1:1 molar ratio. Protein exchanges were visualized using a Nikon A1 Laser Scanning Confocal Imaging System (NIKON Eclipse-TE2000-A1, France) during 30 min. γ44-gliadin-TRITC and γ44-gliadin-FITC were excited respectively at 532 and 488 nm, while emitted lights were recorded at 580 and 525 nm. Confocal images were acquired with a x40 objective (water immersion) and analysed by NIS-Elements AR 3.2 software.

### Droplet formation with increasing NaCl concentration, pH and reducing agent

For assays at 30% ethanol (v/v), **a**ll γ44-gliadin samples were prepared at 4 mg/mL or 104 µM and filtered as previously described. For NaCl assays, γ44-gliadin solutions were first solubilized in MOPS buffer (50 mM, pH 7.2) containing 55% ethanol (v/v) and different NaCl concentrations: 25, 50, 100 or 500 mM. For pH experiments, γ44-gliadin samples were first prepared at different pH values (5.5, 6 and 7.2) in 50 mM MES buffer, 55% ethanol (v/v) and NaCl. Note that for all pH conditions, assays were done at constant ionic strength (48 mM). For experiments in reducing conditions, γ44-gliadin was prepared in MOPS buffer (50 mM pH 7.2, 25 mM NaCl, 55% ethanol (v/v)) with or without 10 mM of DTT. After γ44-gliadin solubilization, all tests were performed by reducing the ethanol concentration from 55 to 30% (v/v) by dilution.

For assays at 0% ethanol, γ44-gliadin samples were prepared in MOPS buffer (50 mM pH 7.2) at different NaCl concentrations: 25, 50, 100 or 500 mM.

All samples were observed under a phase-contrast microscope at the magnification x40. After 30 minutes of centrifugation at 15 000 *g*, supernatants were used to determine solubilized protein concentration using the microplates spectrophotometer as aforementioned. All measurements were performed in triplicate.

### DTNB assay

The free thiol content of non-reduced and reduced γ44-gliadin was determined using the DTNB (5,5′-dithio-bis-(2-nitrobenzoic acid)) assay^53^. γ44-gliadin samples were prepared in MOPS buffer (50 mM pH 7.2, 25 mM NaCl, 55% ethanol (v/v)) with or without 10 mM of DTT as previously described. Protein samples (13 µM) were then incubated with 180 µM of DTNB in thiol assay buffer (50 mM pH 8.0, 25 mM NaCl, 55% ethanol (v/v)) at room temperature for 15 minutes. The 2-nitro-5-thiobenzoic acid generated by the reaction was then detected by its absorbance at 412 nm (ε = 14,150 M^−1^ cm^−1^). Measurements were performed in triplicate.

## Supplementary information

Supplementary information

## Data Availability

The authors declare that all data supporting the findings of this study are available within the article and Supplementary Information, or are available from corresponding authors upon request.
