# Supplementary material for: Role of protein conformation and weak interactions on γ-gliadin liquid-liquid phase separation
Source: Sci Rep. 2019 Sep 16;9:13391. doi: 10.1038/s41598-019-49745-2 (PMC6746847; doi:10.1038/s41598-019-49745-2)
Supplement: Supplementary file 1 — Supplementary information [file 41598_2019_49745_MOESM1_ESM.pdf]

Title: "Role of protein conformation and weak interactions on γ-gliadin liquid-liquid phase separation"

Author(s): Line Sahli, Denis Renard , Véronique Solé-Jamault, Alexandre Giuliani and Adeline Boire.

1- Sequence analysis of γ-gliadin accessions from UniprotKB

To highlight the low variability of γ-gliadin sequences, we proceed to a sequence comparative analysis by using IUPred, ExPASY and CIDER tools. All protein sequences accessions found in UniprotKB with sequence identity close to 100% of γ-gliadin were submitted to analyses (total of four). As previously shown, presence of two distinct domains, one hydrophilic, predicted to be disordered (N-terminal), and one hydrophobic, predicted to be ordered (C-terminal), are established (**Figure S1A**). Despite their distinct molecular weight, no profil difference are observed between the four γ-gliadin accessions. According to CIDER tool, all γ-gliadin accessions are weakly charged proteins predicted to be globular or tadpole (**Figure S1B**). The high content of proline is also noticed in addition to the low net charge per residue (close to zero) (**Figure S1C**). Finally, all these sequence analyses are in agreement with litterature data and tend to prove that there is no noticeable difference between γ-gliadin sequences. γ-gliadins are known to be a group of highly polymorphic proteins, distinguishable by their 2D electrophoretic mobility.

A

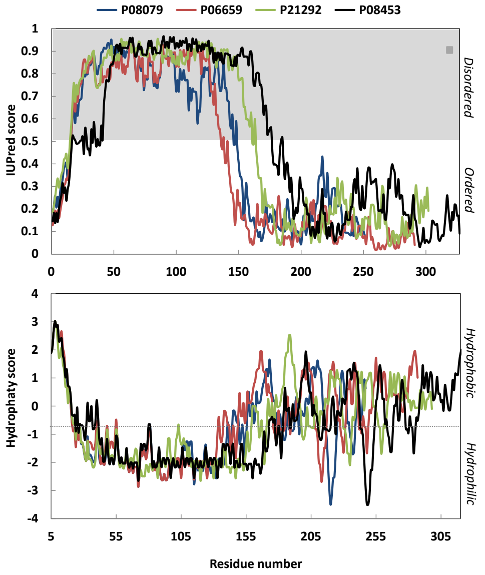

B

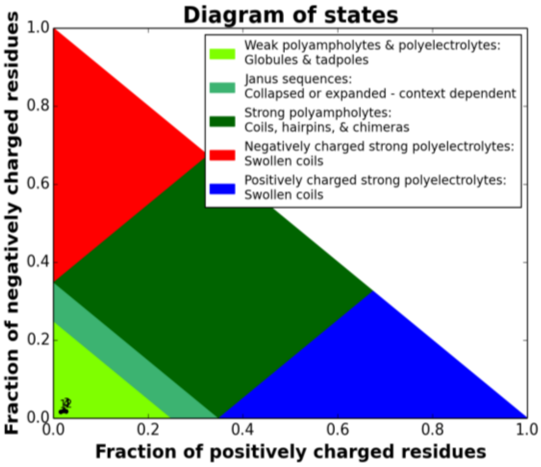

C

ID	Length	κ	FCR	NCPR	Hydropathy	Disorder promoting	Plot region	Warning
1	251	0.330	0.032	0.000	3.651	0.701	1	Fraction of proline is 0.16 [high]
2	291	0.233	0.045	0.010	3.977	0.674	1	Fraction of proline is 0.15 [high]
3	302	0.201	0.043	0.003	3.819	0.689	1	Fraction of proline is 0.17 [high]
4	327	0.216	0.040	0.003	3.796	0.703	1	Fraction of proline is 0.17 [high]

Figure S1: Comparative sequence analysis of γ-gliadin accessions from UniprotKB, related to **Figure 1. A.** *(Top)* IUPred plot predicts the intrinsic disorder of γ-gliadins. Residues with a score above 0.5 are predicted disordered (grey area), and residues with a score below 0.5 are predicted to be ordered (white area). (*Bottom*) Kyte & Doolittle plot estimates hydropathy scores of γ-gliadins residues. Residues with positive scores are predicted hydrophobic while residues with negative scores are predicted hydrophilic. **B.** Diagram of states for IDPs from CIDER tool. All γ-gliadin accessions are located in the region 1 (black points overlapped) corresponding to weak polyampholytes or weak polyelectrolytes that form globule or tadpole-like conformations (light green). **C.** Sequence properties of γ-gliadin accessions. K: charge patterning parameter; FCR, fraction of charged residues; NCPR, net charge per residue which is the difference between the fractions of positively charged and negatively charged residues.

2- Determination of saturation concentrations by fluorescence method

To determine saturation concentrations by fluorescence intensity, a calibration curve of labelled γ44 (fluorescence intensity vs γ44-TRITC concentration) was established at 55% ethanol to ensure complete solubilization of the protein (**Figure S1,** *left*). After inducing liquid-liquid phase separation, fluorescence intensities of dilute phases were measured and concentrations were determined according to the calibration curve. Labelling with TRITC that could affect saturation concentrations of γ44 was also tested (**Figure S1,** *right*). Results show limited effects of the labelling on the saturation concentrations. Saturating concentrations determined by fluorescence measurements are overall consistent with the established phase boundary of the γ44-gliadin phase diagram excepted at 30 % and 35 % ethanol (v/v). At these ethanol proportions, samples are highly turbid which may explain the overestimation of C_{sat} values. All measurements were performed in triplicate and data were expressed as the mean ± standard deviation (SD).

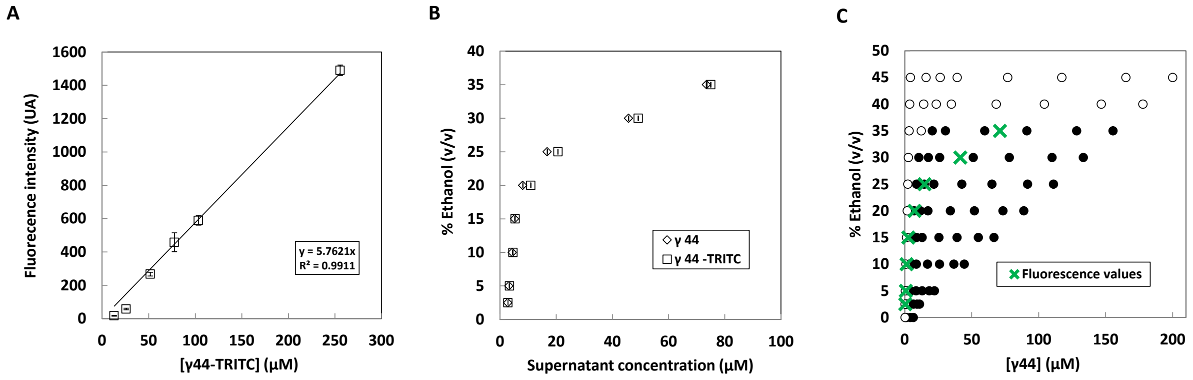

Figure S2: Determination of the saturation concentrations by fluorescence, Related to **Figure 4. A.** Calibration curve of γ44-TRITC (fluorescence intensity vs concentration of γ44-TRITC) in 50 mM MOPS pH 7.2, 25 mM NaCl and 55% of ethanol (v/v) (n=3).**B.** Comparison of saturation of γ44 and γ44-TRITC in 50 mM MOPS pH 7.2, 25 mM NaCl (n=3). **C.** γ44-gliadin non-equilibrium phase diagram with addition of fluorescence values (λ_{ex}~ 555 nm and λ_{em}~ 580 nm) (green crosses).

3- Dynamic behaviour of γ44 liquid-like droplets

To determine whether γ44 droplets are in equilibrium with the diluted phase, we monitored exchanges assays under confocal microscopy at 20% ethanol (v/v). γ44 was previously labelled using two different fluorescent dyes: TRITC (red) or FITC (green). Dynamic exchanges were followed during 30 min at room temperature. The whole microscopic images are shown and demonstrate the progressive inclusion of γ44-FITC (green) into previously formed γ44-TRITC (red) droplets (**Figure S2**).

Fluorescence intensity and exchange rate vary from one droplet to another, some saturated signals are observed in the early stage of the assay (10 min) before becoming blurred (30 min).

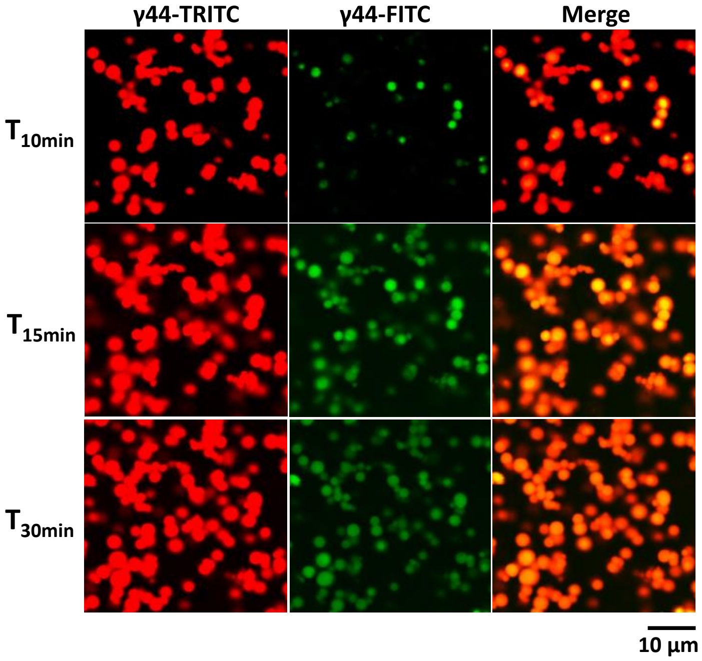

Figure S3: Dynamic and diffusion between the diluted and concentrated phase, Related to **Figure 6.** Entire confocal imaging are reported in this figure. Droplets of γ44-TRITC were pre-formed at 20% ethanol (50 mM MOPS pH 7.2, 25 mM NaCl), during 15 min of equilibration, before adding γ44-FITC (molar ratio 1:1) (47 µM of total protein concentration). The assay was monitored at temperature in time: 10, 15 and 30 min.

4- Analysis of sulfhydryl groups of non-reduced and reduced γ44-gliadin

The free thiol content of non-reduced and reduced (10 mM DTT) γ44-gliadin was quantified using the DTNB (5,5'-dithio-bis-(2-nitrobenzoic acid)) assay at 55% ethanol (pH 8.0). The results show a free thiol content about 300 times higher in presence of 10 mM DTT. In other words, about 75% of the protein disulfide bonds are reduced. These observations are expected and confirm the presence of disulfide bounds in γ-gliadin protein.

γ44-gliadin	Non-reduced	Reduced
Mole of free thiol per mole of protein	0.1 ± 0.002	28.8 ± 0.021

Table 1 : DTNB analysis of free thiols in non-reduced and reduced γ-gliadin. All data are expressed as the mean ± standard deviation (SD).

5- SDS-PAGE (full gel)

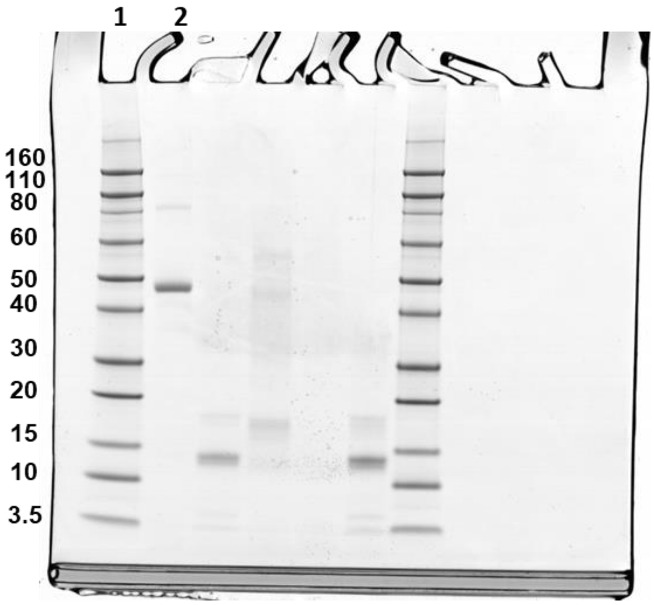

Figure S4: Gel of the γ-gliadin 44 (300 dpi), lane 1 (molecular weight) and 2 (γ44-gliadin) from the left. The other lanes do not concern this work.
